# Loss of GABA_B_ receptor 1 signaling facilitates activation of disease‐associated microglia in the mouse model of Alzheimer’s disease

**DOI:** 10.1002/alz.084480

**Published:** 2025-01-03

**Authors:** JIALI LI

**Affiliations:** ^1^ The Neuroscience Institute at JFK Medical Center, Edison, NJ, USA; Hackensack Meridian School of Medicine, Nutley, NJ USA

## Abstract

**Background:**

Disease‐associated microglia (DAM), which cluster around Aβ plaques, represent a significant pathological hallmark of Alzheimer’s disease (AD) and play a complex role in influencing neuroinflammation, mediating synapse loss, and participating in the phagocytic clearance of Aβ. Nonetheless, the precise mechanisms by which microglial activation extends beyond the traditional M1 and M2 classifications, encompassing a diverse spectrum of states, especially for DAM, closely intertwined with physiological and pathological conditions under Alzheimer’s circumstances remain elusive.

**Method:**

Here, we first combined biochemical techniques and bioinformatic analysis to test and quantify the expression of GABA_B_R1 in both human and mouse AD models. Next, primary microglial cultures, siRNAs, immunohistochemistry, EEG recording and behavior tests were also used for *in vitro and in vivo* tests of loss of GABA_B_R1 signaling in DAM activation, AD‐like pathology, and sleep impairment in this study.

**Result:**

Firstly, we identified that the mRNA expression and protein levels of GABA_B_ receptor 1 (GABA_B_R1) deceased in microglia in the AD brain, especially in DAM compared to resting microglia. Next, we found that Aβ treatment directly lowered GABA_B_R1 expression in microglia. We further found that knocking down GABA_B_R1 in microglia decreased *Trem2* expression, which increases the risk of Alzheimer’s pathogenesis. On the contrary, activating GABA_B_R1 in microglia increased C1qb levels. Notably, the administration of 3xTg mice with the GABA_B_R1 agonist cerebrolysin significantly not only restored microglial shape in the mPFC and hippocampus, with fewer branches but also reduced Aβ and hyperphosphorylation of tau levels in the mPFC and hippocampus and improved behavioral deficits and sleep impairment in AD mice.

**Conclusion:**

Our findings delineate a novel role of loss of the GABA_B_R1 signaling in AD‐associated DAM activation, and elevating GABA_B_R1 activity within DAM holds promise for enhancing microglial structure and function, ultimately alleviating AD‐like pathology and behavioral deficits in the mouse model of Alzheimer’s disease.

